# Triarylborane-“Click” Fluorescent Tag for Orthogonal Amino Acid Labelling, Interactions with DNA, Protein, and Cyclodextrins

**DOI:** 10.3390/ph16091208

**Published:** 2023-08-25

**Authors:** Marta Jurković, Matthias Ferger, Isabela Drašković, Todd B. Marder, Ivo Piantanida

**Affiliations:** 1 Division of Organic Chemistry and Biochemistry, Ruđer Bošković Institute, Bijenička Cesta 54, 10000 Zagreb, Croatia; marta.koscak@irb.hr; 2 Institut für Anorganische Chemie, and Institute for Sustainable Chemistry & Catalysis with Boron, Julius-Maximilians-Universität Würzburg, Am Hubland, 97074 Würzburg, Germany; matthias.ferger@uni-wuerzburg.de; 3 Division of Molecular Biology, Ruđer Bošković Institute, Bijenička Cesta 54, 10000 Zagreb, Croatia; isabela.pehar@irb.hr

**Keywords:** triarylborane, “click”, DNA sensing, protein sensing, fluorescence, cyclodextrin binding

## Abstract

The innovative design of a triarylborane (TB)-dye with one NMe_2_-alkylated (propargylated) group and one NMe_2_ group yielded a system that is both an NMe_2_ π-donor and an inductive NMe_2_-alkyl cationic acceptor. Consequently, the new TB-dye was highly sensitive to a “click” reaction with an azide-substituted lysine side chain (yielding TB-lysine), resulting in a bathochromic shift of emission of 100 nm. In addition, fluorene attached to the lysine C-terminus showed FRET with the TB-chromophore, also sensitive to interactions with targets. Both the TB-dye and TB-lysine showed high affinities towards both DNA and proteins, reporting binding by an opposite fluorimetric response for DNA/RNA (quenching) vs. BSA (increase). Thus, the novel TB-dye is an ideal fluorimetric probe for orthogonal incorporation into bio-targets by “click” reactions due to fluorescence reporting of the progress of the “click” reaction and further sensing of the binding site composition. The TB-dye is moderately toxic to human cell lines after 2–3 days of exposure, but efficiently enters cells in 90 min, being non-toxic at short exposure. The most important product of the “click” reaction, TB-lysine, was non-toxic to cells and showed equal distribution between mitochondria and lysosomes. Further studies would focus particularly on the very convenient monitoring of the progress of “click” conjugation of the TB-dye with biorelevant targets inside living cells by confocal microscopy.

## 1. Introduction

Fluorophores, which can be attached to side chains of amino acids, peptides, or proteins by “click” reactions, have allowed immense advances in biomedical research within the last two decades [1,2]. Consequently, a vast number of already established emissive dyes were adapted to “click” reactions, allowing fine tuning of spectral properties for various applications, including multicolor detection, FRET pair design, and many more [3]. However, there are currently very few low-molecular-mass fluorescent probes available that can bind to both DNA/RNA and proteins with high affinities and differentiate between them by their emissive responses.

To achieve this goal, a pyrene-“click”-peptide-cyanine system [4] was devised, which efficiently interacted with both ds-DNA and BSA, but with different sides of the molecule: cyanine interacted only with DNA and pyrene only with BSA, and the emission response of both fluorophores was proportional to the corresponding target in DNA/BSA mixtures. However, its affinity for DNA or BSA was in the micromolar range, and such a system was not suitable for orthogonal “click” tagging of an amino acid side chain. Much more promising were bis-triarylborane (TB) cations, a novel series of dyes devised only recently, which bound much more strongly to DNA and BSA (10 nM affinity) [5,6,7], allowed distinction between protein and DNA by a 100 nm shift in their emission, and were successfully applied in living cells [8]. Still, these promising dyes interacted only non-covalently, so a “click” ready triarylborane dye was prepared and applied to the construction of amino acid conjugates for sensing the enzymatic activity of DPP3 [9]. However, this type of TB dye was neutral, thus not interacting with DNA.

**Scheme 1:**
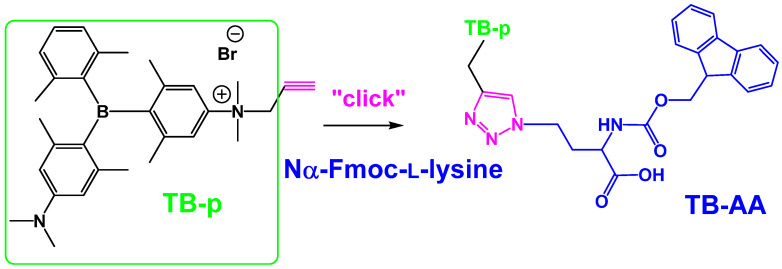
Structures of compounds studied: “click-ready” cationic **TB-p** and amino acid side chain clicked **TB-AA**.

Herein, we present the design and synthesis of a novel, cationic **TB-p** chromophore equipped with a propargyl moiety (Scheme 1) and thus ready for “click” attachment to the azide-side chain of an amino acid and bearing an intrinsic permanent positive charge to insure interactions with ds_DNA/RNA. To prepare a model system, the new TB dye was “clicked” to the amino acid to give conjugate **TB**-**AA** (Scheme 1). Both **TB-p** and the **TB**-**AA** conjugate were characterised in aqueous media and evaluated for interactions with model biomacromolecules: ds-DNA, ds-RNA, and protein (BSA).

In addition, interaction with cyclodextrins of various sizes was characterised because cyclodextrins are widely used as solubilizing agents as well as antidotes, for instance, because of their capacity to bind different small molecules [10,11,12].

For these reasons, Sugammadex (γ-CD-analogue), a registered drug prescribed for the reversal of block anesthesia, is a great example of an antidote [13,14]. In general, plenty of data have demonstrated the cyclodextrin’s interactions of **TB**-**AA** with α-CD, β-CD, and γ-CD were studied to determine the impact of the CD-ring size on **TB**-**AA** affinity complexation.

## 2. Results and Discussion

### 2.1. Spectrophotometric Properties of TB-p and TB-AA

**Figure 1:**
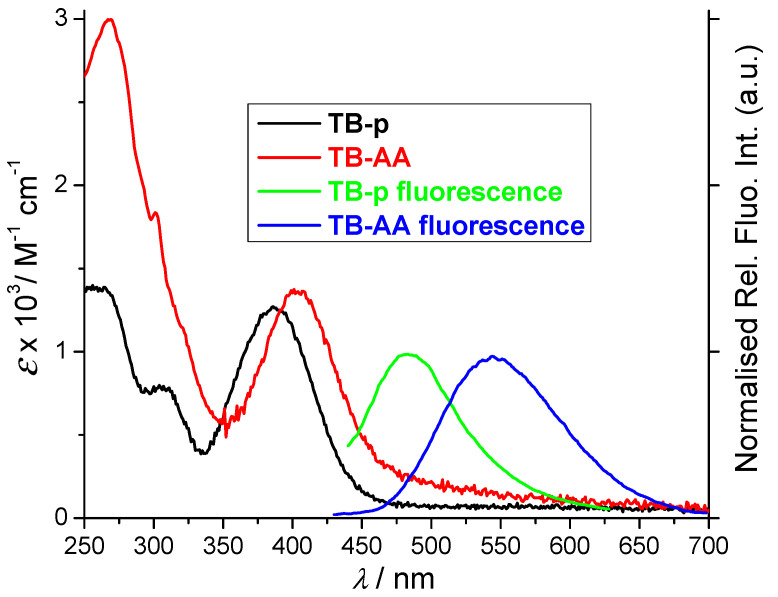
UV/Vis (black and red line) and fluorescence spectra (green and blue line, λ_exc_ = 407 nm, normalised to the emission maximum) of **TB-p** and **TB-AA**. Performed at pH = 7.0, buffer Na cacodylate, *I* = 0.05 M.

The compounds **TB-p** and **TB-AA** are moderately soluble in water; therefore, stock solutions in DMSO (*c* = 0.01 M) were prepared, which were stable at 4 °C for a long period, and subsequently diluted small aliquots in buffer prior to further experiments. The DMSO content was <0.1%. The absorbances of **TB-p** and **TB-AA** aqueous solutions were proportional to their concentrations up to *c* = 1.8 × 10^−5^ M (Supplementary Materials, Figures S1 and S2). Both studied compounds exhibit fluorescence emission, with intensity proportional to the concentration up to around *c* = 3 × 10^−6^ M (Supplementary Materials, Figures S3 and S4). Excitation spectra agree well with UV/Vis spectra. The spectrophotometric properties of the studied compounds are summarised in Figure 1 and in Supplementary Materials, Table S1.

Analysis of spectrophotometric properties (Figure 1) clearly showed a significant difference, with the absorption and particularly emission of **TB-AA** being strongly bathochromically shifted in respect to parent **TB-p**. Mostly, the differences can be attributed to the conversion of the propargylic substituent into triazole, which changed the impact of a quaternary amine substituent on the TB chromophore. Also, the increase in **TB-AA** absorbance in the 250–350 nm range compared with **TB-p** can be attributed to the two additional chromophores, fluorene and triazole.

It should be stressed that **TB-p** and **TB-AA** emission spectra are strongly solvatochromic. Particularly, **TB-p** is unique with regard to previously studied triarylboranes [5,6,7,8,9], in that it has both an NMe_2_ π-donor and an inductive NMe_2_-alkyl cationic acceptor, the latter making the boron a better π-acceptor. This makes such a chromophore highly sensitive to substituents (as denoted above for the propargyl-to-triazole change) as well as to changes in the microenvironment. The latter property would hopefully sense differences in binding sites within DNA/RNA vs. binding sites in proteins (BSA), thus making, e.g., **TBAA** a selective reporter upon binding to both targets.

In addition, **TB-AA** contains two fluorophores (TB and fluorene), which both have absorption bands at 270–300 nm, whereby fluorene emits fluorescence at 300–450 nm (Supplementary Materials, Figure S5), overlapping with the absorbance peak of TB (Figure 1) and therefore allowing FRET contribution to the TB emission at 550 nm. Indeed, **TB-AA** emission strongly depends on the excitation wavelength (Supplementary Materials, Figure S5): if excited at 270 nm, emission at 550 nm will represent TB emission with an additional fluorene-FRET component, and if excited at 407 nm, fluorescence will represent the TB emission only (Figure 1). Intriguingly, under the same experimental conditions (λ_exc_ = 270 nm), **TB-p** does not emit measurable fluorescence (Supplementary Materials, Figure S5), which again supports FRET from fluorene to TB fluorophore in **TB-AA**.

### 2.2. Non-Covalent Interactions of TB-p and TB-AA with ds-DNA, ds-RNA, and BSA

**Figure 2:**
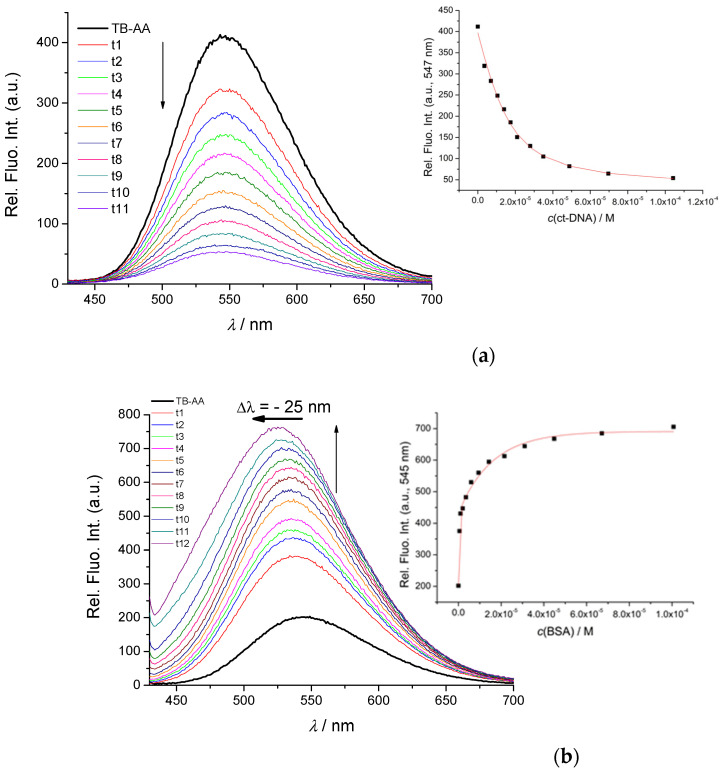
(**a**) Fluorimetric titrations of **TB-AA** (*c* = 3 × 10^−6^ M; λ_exc_ = 407 nm) with (**a**) ctDNA and (**b**) BSA. Inserts show dependence of emission intensity at λ_max_ = 547 nm on c(polynucleotide), (▬) data fit to Scatchard Equation [15,16]. Performed at pH = 7.0, buffer sodium cacodylate, *I* = 0.05 M.

The addition of ds-DNA/RNA to **TB-p** and **TB-AA** generally quenched their emission (Figure 2a, Supplementary Materials, Figures S6 and S7), in line with previously observed changes for the TB-analogues with aliphatic linkers or substituents [5,6,7]. Titration data were obtained by non-linear regression fitted to the Scatchard equation [15,16], yielding the binding constants (Table 1). The analysis of the obtained results (Table 1) revealed that the intensity of emission quenching was much stronger for **TB-AA**, although binding constants were similar, suggesting that the higher sensitivity of **TB-AA** fluorophore to DNA/RNA can be attributed to the change in electronic properties of TB upon “click” conjugation (Figure 1 and related discussion) and not increased binding affinity.

The addition of protein BSA resulted in a strong increase in **TB-p** and **TB-AA** emission (Figure 2b), with titration data fitting nicely to the 1:1 stoichiometry of the dye/BSA complex. Thus, compounds bind to the BSA with biologically relevant 10 µM affinity (Table 1), likely in one of the binding sites for small molecules [17].

Further, to see the impact of binding to ds-DNA on the efficiency of FRET in **TB-AA**, the excitation of **TB-AA** at λ_exc_ = 300 nm was applied, at which fluorene is still excited but DNA does not absorb excitation light. The addition of the large excess of ct-DNA (to ensure almost full formation of a **TB-AA**/ct-DNA complex) decreased the emission at 550 nm (Supplementary Materials, Figure S8), similarly to the titration with only TB-excitation (Figure 2a). Thus, although FRET from fluorene to TB in **TB-AA** is still efficient, binding to DNA partially quenches TB emission by a set of non-covalent interactions that are increasing the non-radiative relaxation of the TB chromophore.

To conclude, the emission of the new cationic TB-chromophore, due to its complex nature as both an NMe_2_ π-donor and an inductive NMe_2_-alkyl cationic acceptor (as discussed above), is highly sensitive to the microenvironment, as demonstrated by the opposite emission changes of **TB-p** or **TB-AA** upon insertion into the DNA/RNA or BSA binding site.

To study the impact of **TB-p** and **TB-AA** on the structural properties of ds-DNA or ds-RNA, two mutually independent methods for monitoring DNA/RNA properties were applied, namely thermal denaturation assays and circular dichroism spectrophotometry.

Namely, ds-polynucleotide solutions, when heated, unwind and disassemble into two single-stranded polynucleotides. At a well-defined temperature (*T*_m_ value), 50% of a particular ds-DNA or ds-RNA sequence is dissociated into corresponding ss-polynucleotides; this temperature is commonly used for the characterisation of various ds-DNA or ds-RNA-related processes. For example, the binding of small molecules usually enhances the thermal stability of the ds-polynucleotides. The enhanced *T*_m_ value compared with the native value of free DNA/RNA gives a positive ∆*T*_m_ value [18], which can be correlated with the various binding modes; however, a final conclusion usually requires confirmation by an independent method [19]. For example, most intercalating agents into ds-DNA cause stabilisation for Δ*T*_m_ > 5 °C due to aromatic stacking interactions with DNA base pairs, whereas the binding of small molecules within the polynucleotide groove, if driven mostly by hydrophobic effects (weak positive charge, no H-boning groups), could have a negligible stabilising outcome [19].

The addition of **TB-p** and **TB-AA** did not influence the thermal denaturation of ds-DNA or ds-RNA (Supplementary Materials, Figures S9–S12, Table S2), in agreement with previously observed TB monocations [7] or TB-“click” conjugates [9].

Further, the circular dichroism (CircD) spectrophotometry can give additional information about the structural properties of the ds- DNA or ds-RNA due to the chiral helicity of polynucleotides. Moreover, if an achiral small molecule, such as **TB-p**, binds uniformly to dsDNA/RNA it can acquire an induced CircDspectrum (ICircD), directly dependent on a dominant binding site within the DNA/RNA helix [20,21]. The **TB-AA**, as a chiral molecule, possessed a CircD spectrum, its intensity being a few orders of magnitude lower than DNA/RNA spectra and thus not interfering with experiments.

**Table 1:** Binding constants (log *K_s_*) and emission changes (^c^ ΔInt) **TB-p** and **TB-AA** with ds-polynucleotides and BSA calculated by processing fluorimetric titrations, performed at pH = 7.0, sodium cacodylate buffer, *I* = 0.05 M.

Compound	^a^ ct-DNA	^a^ poly A–poly U	^b^ BSA
**TB-p**	5.0(^c^ 0.87)	7.1(^c^ 0.51)	4.5(^c^ 47.83)
**TB-AA**	5.9(^c^ 0.13)	6.0(^c^ 0.16)	4.7(^c^ 3.78)

^a^ Processing titration data by means of Scatchard equation [21] gave values of the ratio *n*[bound dye]/[polynucleotide] = 0.1 and 0.3. For easier comparison, all log *K_s_* values were re-calculated for fixed *n* = 0.2. Correlation coefficients were >0.99 for all calculated values of log *K_s_*. ^b^ Best fit obtained for the dye/BSA 1:1 stoichiometry. ^c^ ΔInt = Int (100% complex)/Int_0_.

The addition of both **TB-p** and **TB-AA,** had only a negligible impact on the CircD spectra of ds-DNAs and ds-RNA (Supplementary Materials, Figures S11 and S12), and induced CircD bands > 300 nm were observed. Such a weak impact on the CircD spectra of DNA/RNA and the absence of induced CircD bands were observed for most of the previously studied TB-analogues [5,6,7,8].

Thus, all spectrophotometric studies and thermal denaturation experiments support binding of both **TB-p** and **TB-AA** in the grooves of ds-DNA/RNA, with non-specific orientation of the TB chromophore with respect to the DNA/RNA chiral axis. Due to the only positive charge, the compounds studied do not stabilise DNA/RNA significantly against thermal denaturation; thus, considerably strong binding constants could be attributed to the hydrophobic and Van der Waals interactions within DNA/RNA grooves (Supplementary Materials, Figures S15–S17). This finding agrees with a previous systematic study of the impact of the positive charge of bis-TB compounds on binding affinity towards DNA/RNA and thermal stabilisation [7], which showed an increase in affinity/stabilisation proportional to the increase in positive charge.

### 2.3. Interactions of TB-AA with Cyclodextrins

**Figure 3:**
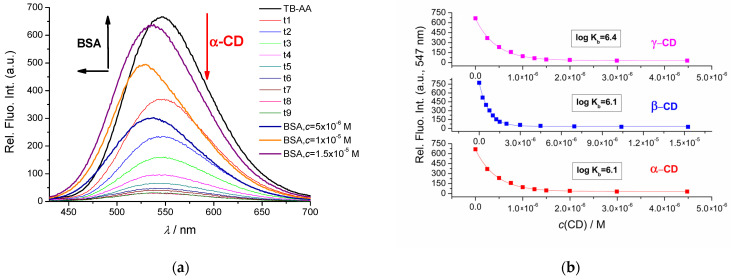
(**a**) Fluorimetric titration of **TB-AA** (*c* = 3 × 10^−6^ M; λ_exc_ = 407 nm) with α−CDs; (**b**) Dependence of **TB-AA** intensity at λ_max_ = 547 nm on *c*(α, β, γ−cyclodextrin), at pH 7.0 in sodium cacodylate buffer, *I* = 0.05 M.

One of the applications of amino acid dyes is encapsulation within supramolecular carriers (in this case, cyclodextrin) to protect them from low-affinity targets and reversible uptake by the final biorelevant target, for which the corresponding amino acid shows high affinity. For this purpose, the complexation of **TB-AA** into cyclodextrins of various sizes was studied. The titration experiments revealed efficient quenching of fluorophore emission and, for all CDs, very similar micromolar affinity, irrespective of CD cavity size (Figure 3, Supplementary Materials, Figure S16). The subsequent addition of BSA efficiently extracted dye from the cyclodextrin cavity, demonstrating the reversibility of binding.

### 2.4. Biological Activity of TB-p and TB-AA

The previous work showed that TB dyes can efficiently enter human cells and accumulate in various intracellular targets while being negligibly toxic [6,7,8,9]. However, any novel dye or conjugate aiming towards applications in bioimaging needs to be tested for antiproliferative activity against the most commonly used cell lines. Also, some TB dyes showed photo-induced cytotoxicity due to the light-sensitisation of singlet oxygen and the development of ROS species [6].

#### 2.4.1. **TB-AA** Exhibits No Cytotoxic Effect Regardless of UV Light Irradiation

**Figure 4:**
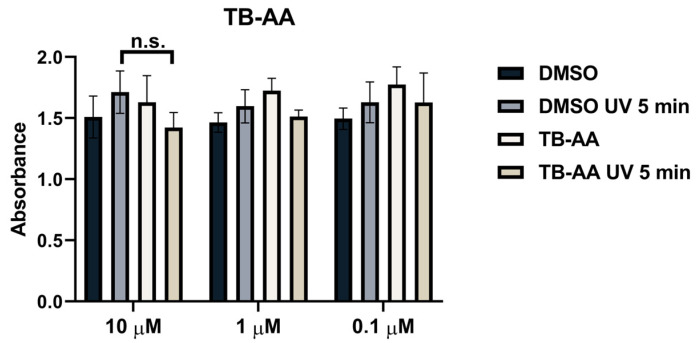
Cell toxicity of **TB-AA** in A549 cells with or without exposure to UV light. Data are presented as absorbance values ± SD resulting from the four replicates. Control samples are cells treated with DMSO in the same dilution as the tested compound. Representative data collected in two independent experiments yielding similar results were averaged and shown.

The cytotoxic effect of **TB-p** and **TB-AA** on the human lung carcinoma cell line (A549) at different concentrations (10 μM, 1 μM, and 0.1 μM) was assessed by the MTT assay. Compound **TB-AA** at all three concentrations did not cause cytotoxic effects in the A549 cell line. The cytotoxic effect did not increase when the **TB-AA** treated cells were exposed to UV light (Figure 4).

**Figure 5:**
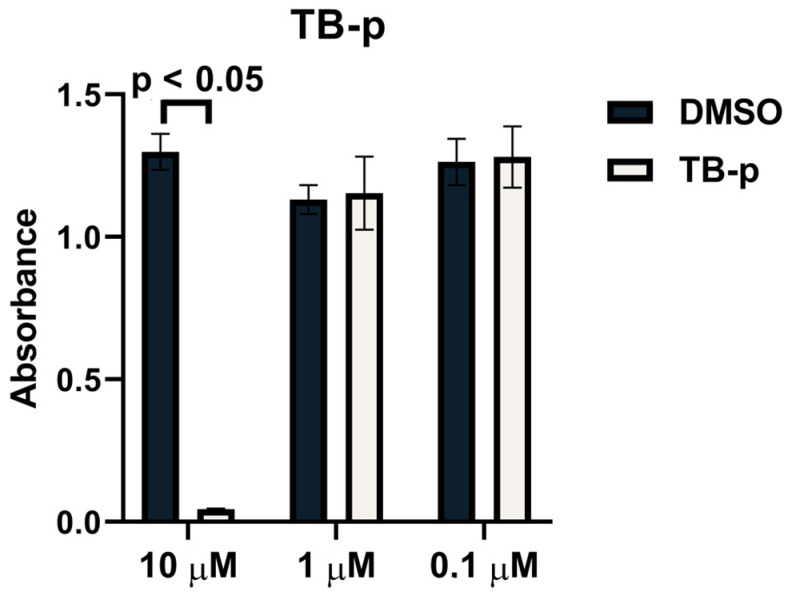
Cell toxicity of **TB-p** in A549 cells. Data are presented as absorbance values ± SD resulting from the four replicates. Control samples are cells treated with DMSO in the same dilution as the tested compound. Data from one biological replica and two independent experiments that yielded similar results are shown.

In contrast to **TB-AA**, **TB-p** caused a considerable cytotoxic effect in the A549 cell line at the highest concentration of 10 µM (Figure 5), whereas at lower concentrations it was negligibly toxic. Since so far all TB dyes studied were non-toxic, it seems that eventually the free propargylic group could induce some minor biological effect. This would limit applications of **TB-p** in living cells; however, this disadvantage could eventually be solved by the application of dibenzocyclooctyne- (DBCO) reagent and the SPAAC “click” reaction [22,23].

#### 2.4.2. Intracellular Localisation of Compounds

**Figure 6:**
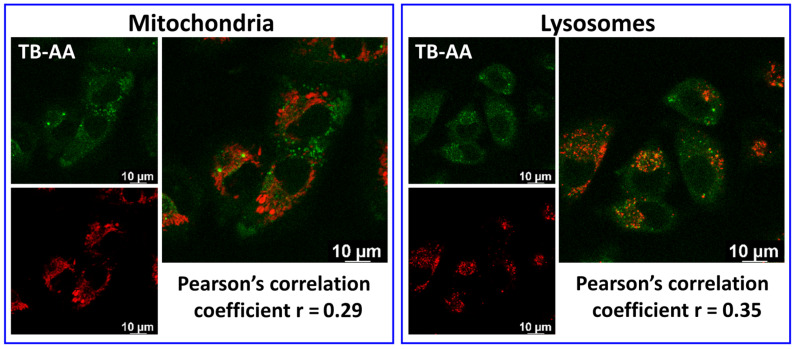
Collocalisation of **TB-AA** (λ_exc_ = 457 nm; λ_em_ = 500–600 nm) with mitochondria (labelled with Mitotracker in red) or lysosomes (labelled with Lysotracker in red) observed by confocal microscopy. Cells were treated with 10 µM of the respective compound for 90 min at 37 °C, and collocalisation was observed in live cells by confocal microscopy. Collocalisation is assessed by the Pearson correlation coefficient. Analysis was conducted using ImageJ software and the appropriate JACoP plugin [24].

The intracellular localisation of **TB-p** and **TB-AA** was assessed by co-localisation experiments using specific stains for different cytoplasmic organelles, namely mitochondria and lysosomes (Figure 6). Thus, cells were treated with **TB-AA**, fixed with PFA, and colocalisation studies were conducted with Lysotracker (a marker for lysosomes) and Mytotracker (a marker for mitochondria). Based on Pearson’s correlation coefficient, the **TB-AA** compound did not show significant collocalisation with mitochondria (Pearson’s coefficient of 0.29), and there was only marginally better collocalisation observed with lysosomes (Pearson’s coefficient of 0.35) (Figure 6). Thus, this particular amino acid conjugate, **TB-AA**, is not organelle-selective.

**Figure 7:**
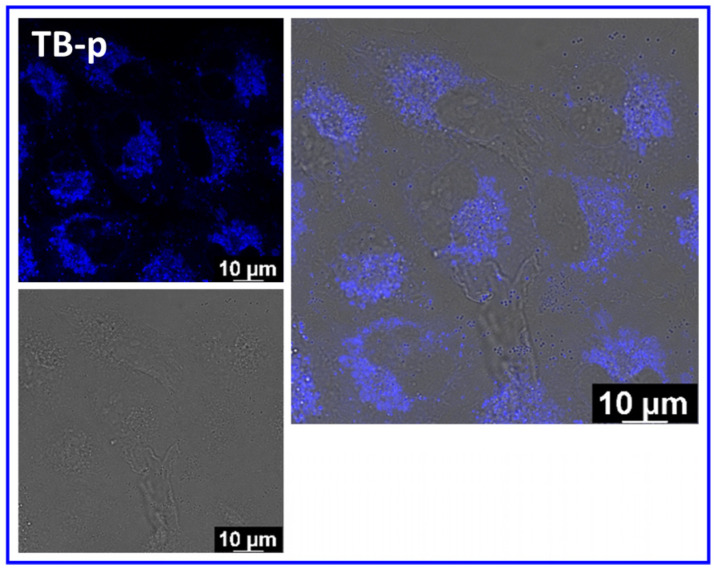
Accumulation of **TB-p** (λ_exc_ = 405 nm; λ_em_ = 450–500 nm) in A549 cells observed by confocal microscopy. Cells were treated with 10 µM of the respective compound for 90 min at 37 °C, and dye presence was observed in live cells by confocal microscopy.

Subsequently, cells were treated with **TB-p** and live cell confocal microscopy was performed to observe the entry of the compound into the cells 90 min after the treatment compound **TB-p** was detected within the cell (Figure 7). Due to weak fluorescence in the blue range of the spectrum, *c*(**TB-p**) =10 µM was used, at which point this dye already exhibited an antiproliferative effect (Figure 5). However, the incubation with dye was only 90 min, while toxicity in the MTT test was noticeable only after 2 days; therefore, it can be estimated that cell penetration and intracellular distribution were not affected by cell deterioration. Cell autofluorescence and weak emission did not allow collocalisation experiments as performed with **TB-AA**.

## 3. Materials and Methods

Synthesis and Characterization. Unless otherwise noted, the following conditions apply. Reactions were performed using standard Schlenk or glovebox (Innovative Technology Inc., Amesbury, MA USA) techniques under an atmosphere of argon. Only oven-dried glassware was used. Solvents used for reactions were HPLC-grade, dried using an Innovative Technology Inc. Solvent Purification System, and further deoxygenated by saturation of the solvent with argon.

*Bis*[4-(N,N-dimethylamino)-2,6-dimethylphenyl]-2,6-dimethylphenylborane was synthesised according to literature procedures [25]. All other starting materials and solvents were purchased from commercial sources and were used without further purification.

Reaction progress was monitored using thin layer chromatography (TLC) plates pre-coated with a layer of silica (Polygram^®^ Sil G/UV254) with fluorescent indicator UV254 from Marchery- Nagel. Automated flash column chromatography was performed using a Biotage^®^ Isolera Four system with silica gel (Biotage SNAP cartridge KP-Sil 50 g or KP-Sil 100 g obtained from Biotage, Uppsalla, Sweden) as the stationary phase and the solvent system indicated. Solvents were generally removed in vacuo using a rotary evaporator at a maximum temperature of 50 °C.

^1^H, ^13^C{^1^H}, and ^11^B{^1^H} solution NMR spectroscopic data were obtained at ambient temperature using a Bruker Avance 500 NMR spectrometer (operating at 500 MHz for ^1^H, 125 MHz for ^13^C{^1^H}, and 160 MHz for ^11^B{^1^H}). Chemical shifts (*δ*) were referenced to solvent peaks as follows. ^1^H NMR spectra were referenced via residual proton resonances of CD_2_Cl_2_ (5.32 ppm). ^13^C{^1^H} spectra were referenced to CD_2_Cl_2_ (53.84 ppm). ^11^B{^1^H} spectra were referenced to external BF_3_**·**OEt_2_.

Elemental analyses were performed on an Elementar vario MICRO cube elemental analyzer. High-resolution mass spectrometry (HRMS) was performed with a Thermo Fisher Scientific Exactive Plus Orbitrap MS System. ASAP measurements were performed with an ACPI source and corona needle at 400 °C, unless otherwise noted.

Synthesis of 2,6-Dimethylphenyl-4-(*N*,*N*-dimethylamino)-2,6-dimethylphenyl-4-(*N*,*N*-dimethyl-*N*-(prop-2-yn-1-yl)ammonium)-2,6-dimethylphenylborane **(TB-p)**

*Bis*[4-(N,N-dimethylamino)-2,6-dimethylphenyl]-2,6-dimethylphenylborane (200 mg, 0.48 mmol, 1.0 eq) was dissolved in acetone (10 mL) and propargylbromide (80 w% in toluene, 0.18 mL, 1.67 mmol, 3.5 eq) were added. The mixture was stirred for 4 d with exclusion of light. The solvent was removed in vacuo without heating. The crude product was dissolved in CH_2_Cl_2_, and impurities were removed by passing the solution through a silica-gel pad and washing with CH_2_Cl_2_. The product was obtained by washing it from the silica-gel pad with CH_2_Cl_2_/MeOH (1/1). Compound **TB-p** was afforded as a colourless solid (190 mg, 75%).

**^1^H NMR** (500 MHz, CD_2_Cl_2_, r.t., ppm): *δ* = 7.14 (s, 2H), 7.13 (m, 1H), 6.92 (m, 2H), 6.32 (m, 2H), 5.50 (d, *J* = 2 Hz, 2H), 3.97 (s, 6H), 2.98 (s, 6H), 2.60 (t, *J* = 2 Hz, 1H), 2.13 (br s, 6H), 2.03 (br s, 6H), 1.96 (s, 3H), 1.89 (s, 3H) (Supplementary Materials Figure S19).

**^13^C{^1^H} NMR** (125 MHz, CD_2_Cl_2_, r.t., ppm): 152.6, 151.7, 147.5, 145.0, 144.6, 144.1, 143.4, 140.8, 140.3, 133.8, 129.6, 128.1, 119.2, 111.9, 80.4, 72.6, 59.4, 54.4, 40.0, 24.2, 24.1, 23.3, 23.1 (Supplementary Materials Figure S20).

**^11^B{^1^H} NMR** (160 MHz, CD_2_Cl_2_, r.t., ppm): *δ* = 75.

**HRMS** (ASAP pos): m/z found: 451.3268 [M-Br]^+^, calcd.: 451.3279 [C_31_H_40_BN_2_]^+^ (|Δ| = 2.44 ppm).

**Elemental analysis:** found: C: 69.80%, H: 7.94%, N: 5.47; calc.: C: 70.07%, H: 7.59%, N: 5.27.

Synthesis of N-((1-(3-(((9H-fluoren-9-yl)methoxy)carbonyl)-3-carboxypropyl)-1H-1,2,3-triazol-4-yl)methyl)-4-((4-(dimethylamino)-2,6-dimethylphenyl)(2,6-dimethylphenyl)boryl)-N,N,3,5-tetramethylbenzenaminium bromide **(TB-AA).**

To a round bottom flask (5 mL), **TB-p** (28 mg; 0.053 mmol) and 2-(((9H-fluoren-9-yl)methoxy)carbonyl)-4-azidobutanoic acid [26] (19.4 mg; 0.053 mmol) were added, as well as 1 mL of a mixture of EtOH/H_2_O (*v*/*v* = 1:1; 1.584 mL). The flask was placed in an ultrasonic bath briefly to enhance dissolution of solids. Then, an aqueous solution of sodium ascorbate (2.10 mg in 146 μL H2O; 0.0106 mmol) and copper (II) sulphate pentahydrate (0.660 mg in 146 μL H2O; 0.00264 mmol) were added. An equal amount of EtOH was added (292 μL) to match total volume of 1.584 mL. The orange-yellow suspension was stirred for 48 h in the dark (wrapped in Al-foil) at room temperature. The reaction was monitored by TLC. The solvent was removed on a rotary evaporator, diluted in a mixture of EtOH and excess CH_3_Cl_2_, and purified by preparative chromatography (CH_2_Cl_2_/MeOH 3:1). The resulting compound was dried under vacuum, yielding the product (25.3 mg, 54%) as a yellow oil solid.

**^1^H NMR** (300 MHz, DMSO) δ 7.87 (s, 3H, CH, triazole, and CH_2_), 7,63 (s, 2H, CH_2_), 7.39–7.30 (m, 8H, 7× CH, and NH), 7.10–7.07 (m, 1H, CH-Fmoc), 6.93–6.90 (m, 2H, 2× CH (Fmoc)), 6.75 (br s, 1H, CH-Fmoc), 6.36–6.30 (6.33 (d, J = 6.33, 17.3 Hz, 2H, 2× CH(Fmoc)), 5.13 (s, 2H, CH_2_), 4.33–4.22 (m, 7H, 3× CH (Fmoc), 2× CH_2_), 3.59 (s, 6H, 2× NCH_3_), 2.01–1.85 (m, 24H, 6× CH_3_, and 2xNCH_3_), 1.23–0.84 (m, EtOH in DMSO) (Supplementary Materials, Figure S21).

**HRMS** (LC/Q-TOF): m/z calcd. for C_50_H_58_BN_6_O_4_ [M-Br]^+^: 816.4649, found: 816.4647 (|Δ| = 0.24 ppm) (Supplementary Materials, Figure S22).

Study of interactions with ds-DNA, ds-RNA, BSA, and Cyclodextrins (CDs): Experiments were conducted in aqueous buffer solution (pH = 7.0 or pH = 5.0, *I* = 0.05 M, sodium cacodylate buffer). The UV-Vis spectra were collected by Varian Cary 100 Bio spectrometer; fluorescence spectra were collected by Varian Cary Eclipse fluorimeter; quantum yield measurements and fluorescence lifetime data were collected by FS5 Spectrofluorometer from Edinburgh Instruments Ltd.; and CircD spectra were collected by JASCO J815 spectropolarimeter. All measurements were conducted at 25.0 °C using appropriate quartz cuvettes (path length: 1 cm).

Polynucleotides were purchased as noted: poly A–poly U (ds-RNA) (Sigma), calf thymus (ct)—DNA (Aldrich), and dissolved in sodium cacodylate buffer, *I* = 0.05 M, pH = 7.0. The ct-DNA was sonicated and filtered through a 0.45 µm filter to obtain mostly short (ca. 100 base pairs) rod-like B-helical DNA fragments. Concentration of DNA or RNA was determined spectroscopically as the *c*(nucleobase) using producer-provided molar extinction coefficients.

BSA, α-CD, β-CD, and γ-CD were purchased from Sigma Aldrich, Darmstad, Germany, and their stock solutions (*c*(CDs) = 0.001 M and *c*(BSA) = 0.001 M) were prepared in a cacodylate buffer.

Spectrophotometric and fluorimetric titrations were conducted by collecting the spectrum of a dye and then consecutively titrating with small aliquots (1–30 µL) of DNA, RNA, BSA, or CDs, monitoring the change in spectrum of the dye. Total dilution in titration experiments was >1%, corrected.

Thermal denaturation of ds-DNA, ds-RNA, and their complexes with the compounds studied was performed [18] by monitoring the absorption change at 260 nm as a function of temperature. *T_m_* values are the midpoints of the transition curves determined from the maximum of the first derivative and checked graphically by the tangent method. The Δ*T_m_* values were calculated by subtracting the *T_m_* of the free nucleic acid from the *T_m_* of the complex. Every Δ*T_m_* value here reported was the average of at least two measurements. The error in Δ*T_m_* is ± 0.5 °C.

Circular dichroism (CircD) spectra were collected with a scanning speed of 200 nm/min and with three accumulations averaging. A buffer background was subtracted from each spectrum. CircD experiments were performed by titrating the compound stock solution into the polynucleotide solution (*c* = 2 × 10^–5^ M).

Cells: Commercially available human epithelial lung adenocarcinoma A549 cells (ATCC^®^ CCL-185™) were obtained from the American Type Culture Collection (ATCC) and grown according to the supplier’s instructions. The cells were cultured in Dulbecco Modified Eagle’s Medium (DMEM, Sigma Aldrich, New York, USA) supplemented with 10% foetal bovine serum (FBS, Sigma Aldrich, USA). Incubation was performed in a cell incubator (Thermo Fisher Scientific, New York, USA) at 37 °C and 5% CO_2_ in a humidified atmosphere.

Cell viability assay: To obtain a 10 mM stock solution of the chemical compounds (**TB-p** and **TB-AA**), they were diluted sterilely in an appropriate volume of dimethyl sulfoxide solution (DMSO, Gram-Mol, Croatia). The solutions were stored in the dark at 4 °C. The cytotoxic effects of each compound were assessed on A549 cells using the MTT test [27]. Working solutions were prepared by diluting the DMSO stock solutions in DMEM, ensuring that the DMSO content did not exceed 0.1%. Cells were seeded in 96-well tissue culture plates and treated with **TB-AA** or **TB-p** at concentrations ranging from 10 to 0.1 μM. Cells treated with the same dilutions of DMSO served as the control, while cells treated only with DMEM (10% FBS) served as the negative control. After 72 h, the media was removed, MTT solution was added to each well, and the plate was incubated at 37 °C and 5% CO_2_ for 3 h. The resulting MTT-formazan products were dissolved in DMSO, and their absorbance at 600 nm was measured using a microplate reader.

Effect of UV exposure: Cells were seeded on two plates and treated with the working solutions of **TB-AA** as described above. The plates were incubated at 37 °C and 5% CO_2_ for 90 min to allow the compounds to enter the cells. Subsequently, one plate was exposed to UV light (Luzchem reactor, 420 nm, 8 lamps, total 64 W, dose 50.6 mW × m^−2^; ~18 cm lamp to cell-plate) for 5 min, three days in a row at the same time each day, while the other plate was kept in the dark in the cell incubator and served as the control.

Live cell imaging: For live imaging, A549 cells were seeded in Ibidi imaging cell chambers (Ibidi^®^, Germany) and the next day treated with a 10 μM solution of the respective compound. The cells were incubated in the cell incubator for 90 min to allow the compound to enter the cells. For analysing co-localisation with mitochondria or lysosomes, after incubation with the tested compounds, the cells were rinsed and incubated with 100 nM MitoTracker Deep Red or LysoTracker Deep Red solutions (Invitrogen, Molecular Probes), respectively. The cells were incubated for 20 min at 37 °C, allowing the MitoTracker or LysoTracker dye to enter the cells. After incubation, the medium was replaced with 500 µL of fresh DMEM, and the cells were immediately observed by a Leica SP8 X confocal microscope (Leica Microsystems, Wetzlar/Mannheim, Wetzlar, Germany). The probability of co-localisation was expressed using the Pearson correlation coefficient. Analysis was performed by ImageJ software using the appropriate JACoP plugin [24]. The Pearson correlation coefficient, r, can take a range of values from +1 to −1. A value of 0 indicates that there is no association between the two variables. A value greater than 0 indicates a positive association; that is, as the value of one variable increases, so does the value of the other variable. A value less than 0 indicates a negative association; that is, as the value of one variable increases, the value of the other variable decreases.

## 4. Conclusions

The innovative design of the new triarylborane (TB)-dye **TB-p** with only one NMe_2_-alkylated (propargylated) yielded a system that is both an NMe_2_ π-donor and an inductive NMe_2_-alkyl cationic acceptor, at variance to most of the previously studied cationic TB-chromophores. For that reason, the TB chromophore was highly sensitive to the changes in alkylating substituent, thus strongly sensing the "click" reaction in the process of formation of **TB-AA** by bathochromic shift of emission of ca. 100 nm. In addition, in **TB-AA**, fluorene attached to the lysine C-terminus showed FRET with TB-chromophore, similar to that observed for the carbazole-fluorene/TB-Iridium complex [28], with the advantage that interaction of **TB-AA** with the target (ds-DNA) did not completely abolish the FRET.

Both **TB-p** and **TB-AA** showed high affinity towards a broad range of bio-targets, reporting binding by the opposite fluorimetric response for DNA/RNA (quenching) vs. BSA (increase). Thus, a novel TB-chromophore is an ideal fluorimetric probe of orthogonal incorporation to bio-targets by “click” reaction [29], first due to reporting the progress of the chemical reaction and further by sensing of the binding site composition.

The **TB-AA** was bound by insertion into cyclodextrins of various sizes with similar affinity. Subsequent addition of BSA efficiently extracted **TB-AA** from the cyclodextrin cavity, as reported by the opposite emission change, demonstrating the reversibility of binding. Thus, **TB-p** can be used for fluorescent marking of molecules/drugs aimed at cyclodextrin inclusion, transportation, and release.

The **TB-p** dye is moderately toxic to human cell lines after 2–3 days of exposure, but efficiently enters cells in a much shorter period of time (90 min). The most important product of the "click" reaction, **TB-AA**, is non-toxic to cells, even under prolonged UV light exposure. The **TB-AA** collocalisation experiments showed a similar distribution between mitochondria or lysosomes.

Based on the presented results, novel TB-chromophore (**TB-p**) is a very promising lead compound for further studies, supporting further studies focusing on methodology modified for the Cu-free SPAAC “click” reaction [30]. Particularly, its strong bathochromic shift of emission upon “click” reaction of ca. 100 nm would be extremely convenient for monitoring progress of “click” conjugation with bio-target inside living cells by confocal microscopy since the weak blue emission would be replaced by intensive green. So-marked bio-targets would then have a small, globular chromophore with highly sensitive emission based on changes in binding sites or microenvironments.

## Data Availability

Data is contained within the article and Supplementary Material.

## Supplementary Materials

The following supporting information can be downloaded at: https://www.mdpi.com/article/10.3390/ph16091208/s1: Figure S1. Dependence of UV/Vis spectra on concentration of **TB-p**; Figure S2. Dependence of UV/Vis spectra on concentration of TB-AA; Figure S3. Dependence of fluorescence excitation and emission spectra on concentration increase of **TB-p** at λ_exc_ = 387 nm and λ_em_ = 496 nm; Figure S4. Dependence of fluorescence excitation and emission spectra on concentration increase of TB-AA at λ_exc_ = 407 nm and λ_em_ = 547 nm; Figure S5. Emission spectra of Fmoc-Ala-OH (*c* = 5 × 10^−7^ M), **TB-p** (*c* = 5 × 10^−6^ M) and TB-AA (*c* = 5 × 10^−6^ M); Table S1. Electronic absorption and emission data of **TB-p** and TB-AA at pH 7, sodium cacodylate buffer, *l* = 50 mM; Figure S6. (a) Changes in fluorescence spectrum of **TB-p** (*c* = 5.00 × 10^−6^ M) on λ_exc_ = 387 nm upon titration with pApU; b) Dependence of **TB-p** intensity at λmax = 501 nm on c(pApU), at pH 7.0, sodium cacodylate buffer, I = 0.05 M; Figure S7. (a) Fluorimetric titrations TB-AA (*c* = 3 × 10^−6^ M; λ_exc_ = 407 nm) with poly A – poly U; (b) Dependence of emission intensity at λ_max_ = 547 nm on c(pApU). Done at pH = 7.0, buffer sodium cacodylate, *I* = 0.05 M; Figure S8. Emission spectra of TB-AA (*c* = 1 × 10^−6^ M) and complex of TB-AA with 10 times excess ct-DNA; Figure S9. (a) Melting curve of ctDNA upon addition *r* = 0.1 and *r* = 0.3 ([compound/[polynucleotide]) of **TB-p** at pH 7.0; (b) first derivation of absorbance on temperature; Figure S10. (a) Melting curve of pApU upon addition *r* = 0.1 and *r* = 0.3 ([compound/[polynucleotide]) of **TB-p** at pH 7.0; (b) first derivation of absorbance on temperature; Figure S11. (a) Melting curve of ctDNA upon addition *r* = 0.1 and *r* = 0.3 ([compound/[polynucleotide]) of TB-AA at pH 7.0; (b) first derivation of absorbance on temperature; Figure S12. (a) Melting curve of pApU upon addition *r* = 0.1 and *r* = 0.3 ([compound/[polynucleotide]) of TB-AA at pH 7.0; (b) first derivation of absorbance on temperature; Table S2. Tm - Valuesa (°C) for different ratios br of **TB-p** and TB-AA added to polynucleotide; Figure S13. CD titration of (a) ctDNA, (b) pApU (*c* = 2 × 10^−5^ M) with **TB-p**; Figure S14. CD titration of (a) ctDNA, (b) pApU (*c* = 2 × 10^−5^ M) with TB-AA; Figure S15. (a) Fluorimetric titration of **TB-p** (*c* = 5.00 × 10^−6^ M) on λ_exc_ = 387 nm with ctDNA; (b) Dependence of **TB-p** intensity at λ_max_ = 498 nm on *c*(ctDNA), at pH 7.0, sodium cacodylate buffer, *I* = 0.05 M.; Figure S16. (a) Fluorimetric titration of **TB-p** (*c* = 5.0 × 10^−6^ M) on λ_exc_ = 387 nm with pApU; (b) Dependence of **TB-p** intensity at λ_max_ = 501 nm on *c*(pApU), at pH 7.0, sodium cacodylate buffer, *I* = 0.05 M; Figure S17. (a) Fluorimetric titration of **TB-p** (*c* = 5.00 × 10^−6^ M) on λ_exc_ = 387 nm with BSA; (b) Dependence of **TB-p** intensity at λ_max_ = 520 nm on *c*(BSA), at pH 7.0, sodium cacodylate buffer, *I* = 0.05 M; Figure S18. Fluorimetric titration of TB-AA (*c* = 3.00 × 10^−6^ M) on λ_exc_ = 407 nm with (a) β-cyclodextrin; (b) γ-cyclodextrin and competition with BSA, at pH 7.0; Figure S19. ^1^H NMR spectrum of **TB-p**; Figure S20. ^13^C{^1^H} NMR spectrum of **TB-p**; Figure S21. ^1^H NMR spectrum of TB-AA; Figure S22. HRMS analysis of TB-AA
