# Supplementary material for: Triarylborane-“Click” Fluorescent Tag for Orthogonal Amino Acid Labelling, Interactions with DNA, Protein, and Cyclodextrins
Source: Pharmaceuticals (Basel). 2023 Aug 25;16(9):1208. doi: 10.3390/ph16091208 (PMC10535762; doi:10.3390/ph16091208)
Supplement: Supplementary file 1 [file pharmaceuticals-16-01208-s001.zip › pharmaceuticals-2552615-supplementary.pdf]

Triarylborane-"click" fluorescent tag for orthogonal amino acid labelling, interactions with DNA, protein and cyclodextrins

Marta Jurković [1], Matthias Ferger [2], Isabela Drašković [3], Todd B. Marder [2*], Ivo Piantanida [1*]

[1] Division of Organic Chemistry and Biochemistry, Ruđer Bošković Institute, Bijenička Cesta 54, 10000 Zagreb, Croatia; M.K. marta.koscak@irb.hr
[2] Institut für Anorganische Chemie, and Institute for Sustainable Chemistry & Catalysis with Boron, Julius-Maximilians-Universität Würzburg, Am Hubland, 97074 Würzburg, Germany
[3] Division of Molecular Biology, Ruđer Bošković Institute, Bijenička Cesta 54, 10000 Zagreb, Croatia; I.D. isabela.pehar@irb.hr;
* Correspondence: I.P. pianta@irb.hr; T.B.M. todd.marder@uni-wuerzburg.de

Contents

1. Spectrophotometric properties 2
2. Non-covalent interactions of TB-p and TB-AA with ds-DNA, ds-RNA, and BSA 7
3. Thermal denaturation experiments 9
4. Circular dichroism (CircD) experiments 11
5. Interactions with DNA, RNA, BSA 12
6. Interactions of TB-AA with cyclodextrins 13
7. NMR and HRMS data 14

1. Spectrophotometric properties

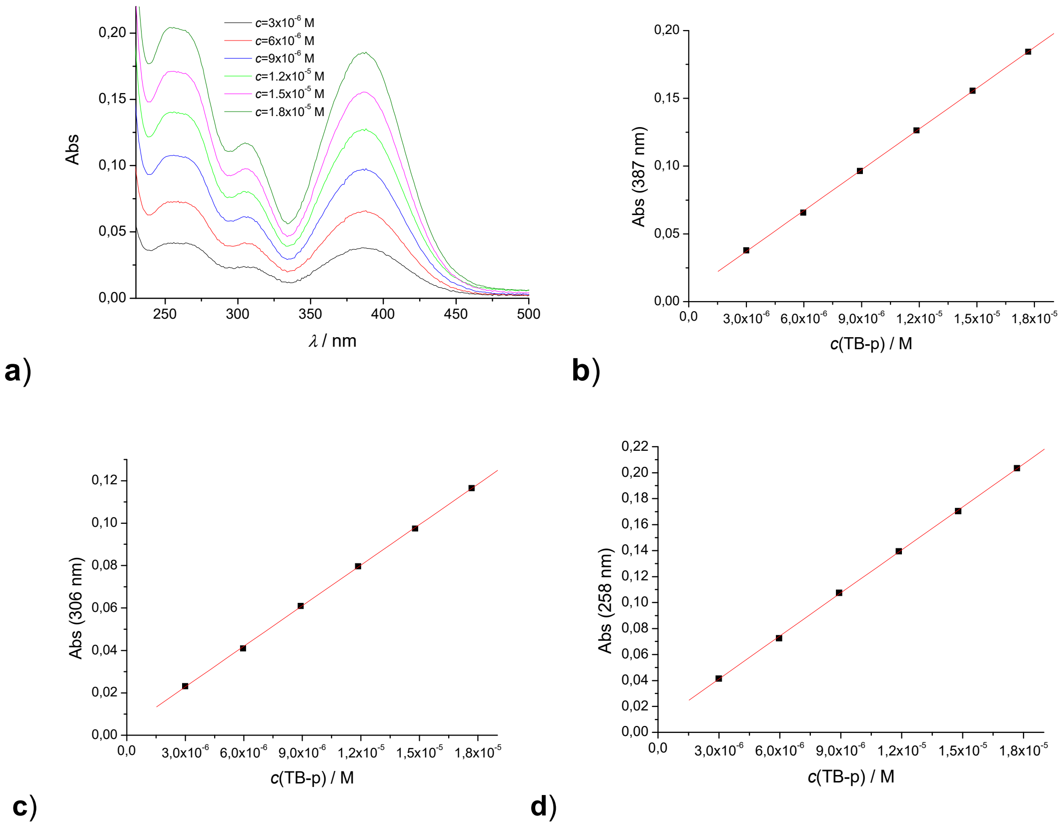

Figure S1. a) Dependence of UV/Vis spectra on concentration of **TB-p**; **b)** Dependence of UV/Vis spectra on concentration increase of **TB-p** (*c* = 2 × 10-5 M) at λ_{max}=387 nm and pH 7.0, sodium cacodylate buffer, *I* = 50 mM; **c**) Dependence of UV/Vis spectra on concentration increase of **TB-p** (*c* = 2 × 10^{-5} M) at λ_{max} = 306 nm and pH 7.0, sodium cacodylate buffer, *I* = 50 mM; **d**) Dependence of UV/Vis spectra on concentration increase of **TB-p** (*c* = 2 × 10^{-5} M) at λ_{max} = 258 nm and pH 7.0, sodium cacodylate buffer, *I* = 50 mM.

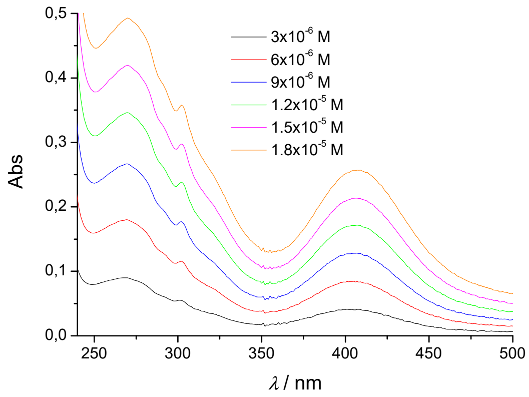

a)

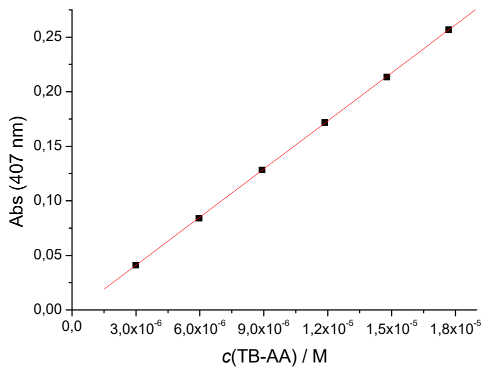

b)

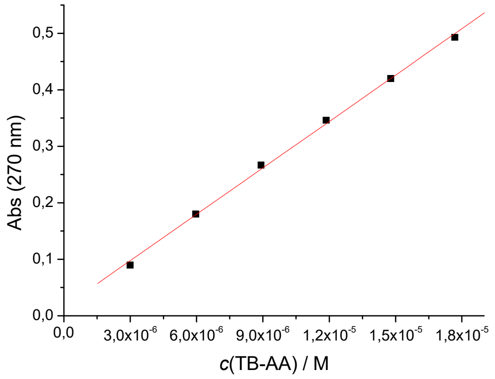

c)

Figure S2. a) Dependence of UV/Vis spectra on concentration of **TB-AA**; **b)** Dependence of UV/Vis spectra on concentration increase of **TB-AA** (c=1.8 × 10^{-5} M) at λ_{max}=407 nm; **c**) Dependence of UV/Vis spectra on concentration increase of **MK-02** (c = 1.8 × 10^{-5} M) at λ_{max} = 270 nm. Done at pH 7.0, sodium cacodylate buffer, I = 50 mM.

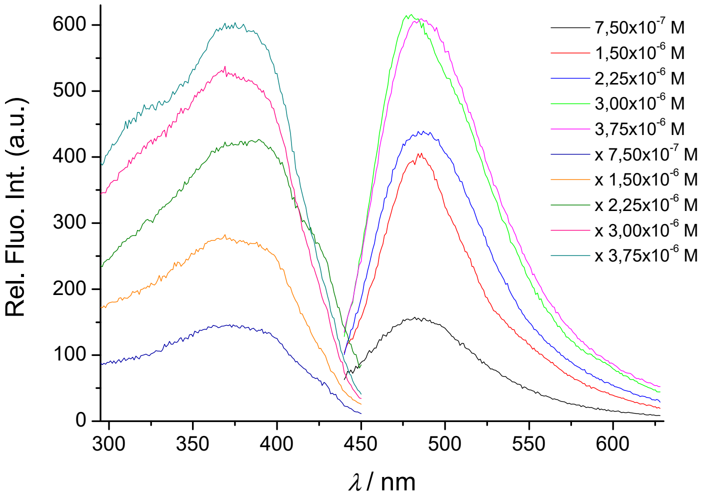

Figure S3. Dependence of fluorescence excitation and emission spectra on concentration increase of **TB-p** at λ_{exc} = 387 nm and λ_{em} = 496 nm. Done at pH 7.0, sodium cacodylate buffer, *I* = 50 mM and with slits 20, 20.

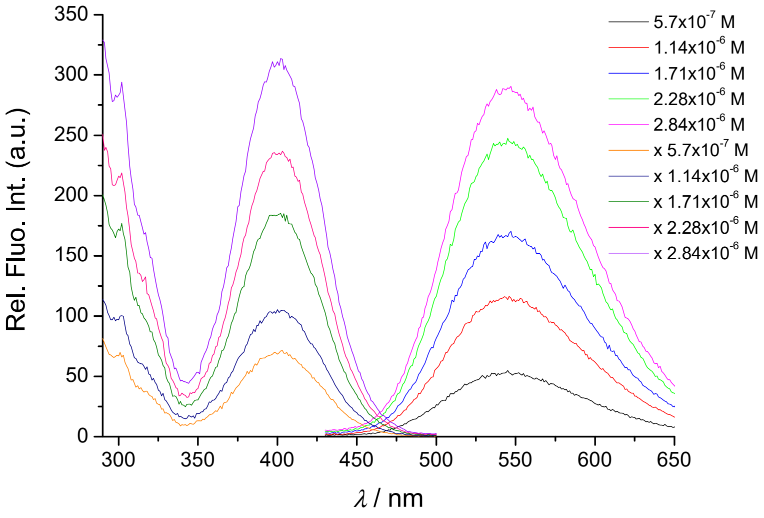

Figure S4. Dependence of fluorescence excitation and emission spectra on concentration increase of **TB-AA** at λ_{exc} = 407 nm and λ_{em} = 547 nm). Done at pH 7.0, sodium cacodylate buffer, *I* = 50 mM with slits 5, 10.

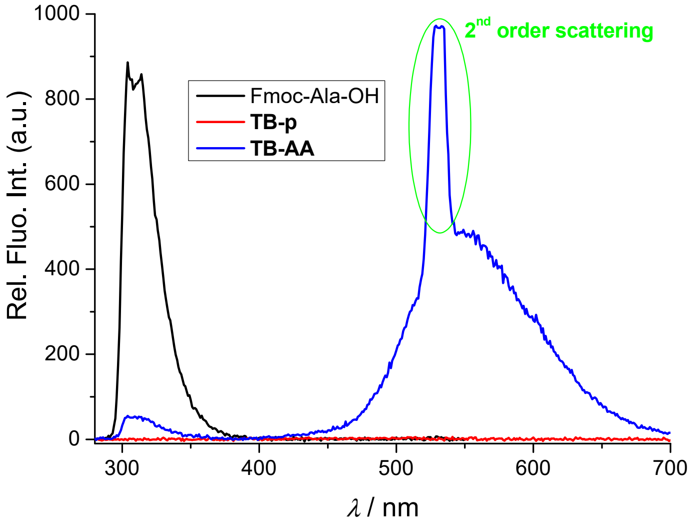

Figure S5. Emission spectra of **Fmoc-Ala-OH** (c=5 × 10^{-7} M)**, TB-p** (c=5 × 10^{-6} M) and **TB-AA** (c=5 × 10^{-6} M) at λ_{exc}=265 nm and slits: 5-5 in sodium cacodylate buffer at pH 7.0, I=50 mM. The baseline of the buffer subtracted from the spectra.

Table S1. Electronic absorption and emission data of **TB-p** and **TB-AA** at pH 7, sodium cacodylate buffer, *I* = 50 mM.

Compound	λ_{max} / nm	ε / mol^{-1} cm^{2}	$^{a}\Phi_{f}$	$^{b}\lambda_{exc}$ / nm	$^{b}\lambda_{em}$ / nm	$^{b}\tau$ / ns	χ2
**TB-p**	387	10036.93	0.15±0.05	405	485	c	c
	306	6371.61					
	258	11037.06					
**TB-AA**	407	14689.87	0.07±0.05	405	547	1.30 (31.82 %)	1.105
	270	27378.65				6.39 (68.18 %)	

[a] The measurements were performed three times and the average values are reported. The associated errors correspond to the maximum absolute deviation. Absolute fluorescence quantum yield was determined by integrating sphere SC-30, Edinburgh Inst., for Argon purged solutions, by λ_{exc} = 387 nm (**TB-p**) / 407nm (**TB-AA**).

[b] Water solutions were purged by Argon, samples were excited by pulsing diodes at 405 nm.

[c] It was not possible to obtain the fluorescence lifetime decay due to inappropriate excitation wavelength.

2. Non-covalent interactions of TB-p and TB-AA with ds-DNA, ds-RNA, and BSA

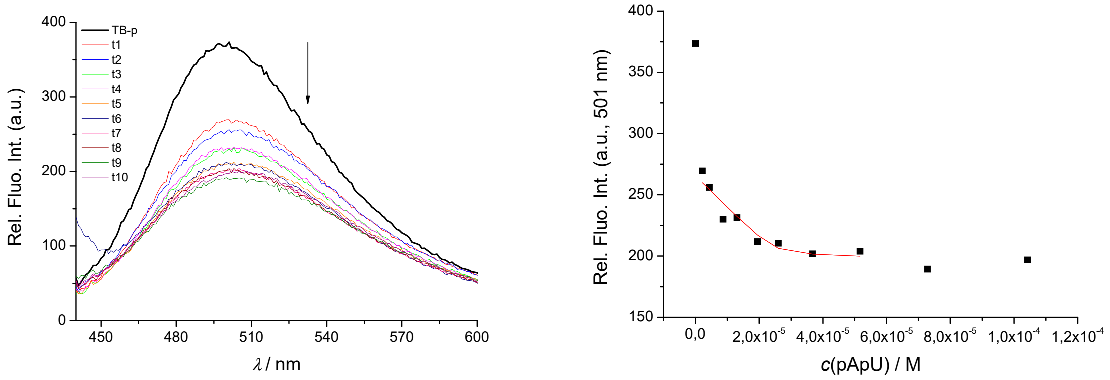

a) **b**)

Figure S6. a) Changes in fluorescence spectrum of **TB-p** (c = 5.00 × 10^{-6} M) on λ_{exc} = 387 nm upon titration with pApU; **b)** Dependence of **TB-p** intensity at λ_{max} = 501 nm on c(pApU), at pH 7.0, sodium cacodylate buffer, I = 0.05 M.

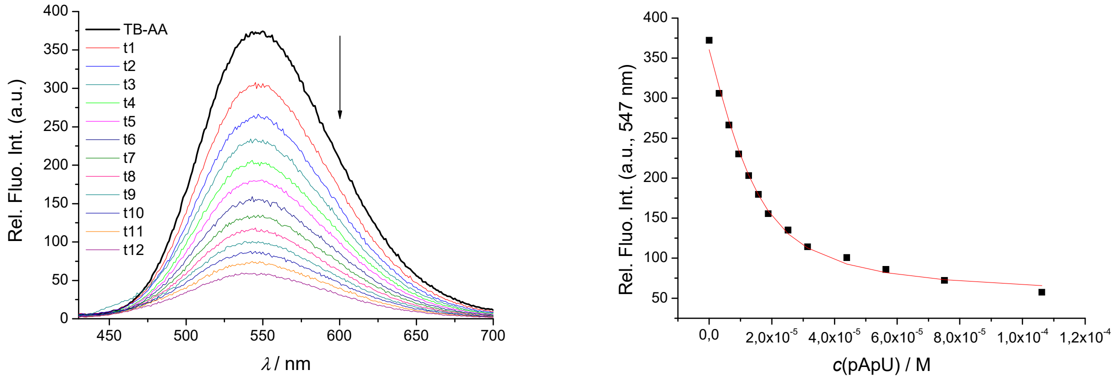

a) **b**)

Figure S7. a) Fluorimetric titrations **TB-AA** (c = 3 × 10^{-6} M; λ_{exc} = 407 nm) with poly A – poly U; **b**) Dependence of emission intensity at λ_{max} = 547 nm on c(pApU). Done at pH = 7.0, buffer sodium cacodylate, I = 0.05 M.

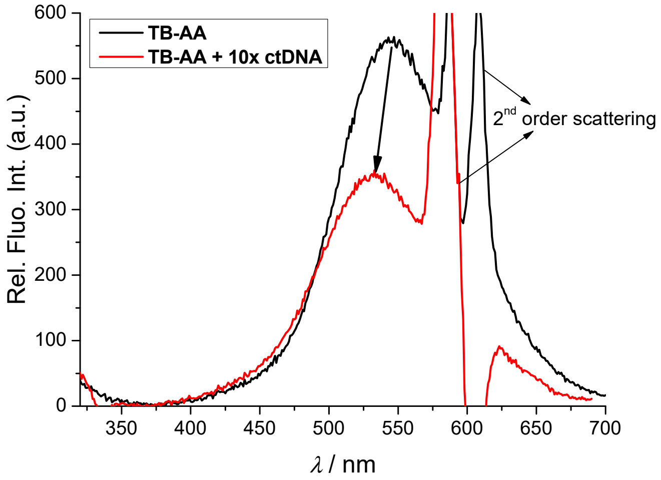

Figure S8. Emission spectra of **TB-AA** (c=1 × 10^{-6} M) and complex of **TB-AA** with 10 times excess ct-DNA, λ_{exc}=300 nm, slits: 10-10. The baseline of the buffer subtracted from the spectra.

3. Thermal denaturation experiments

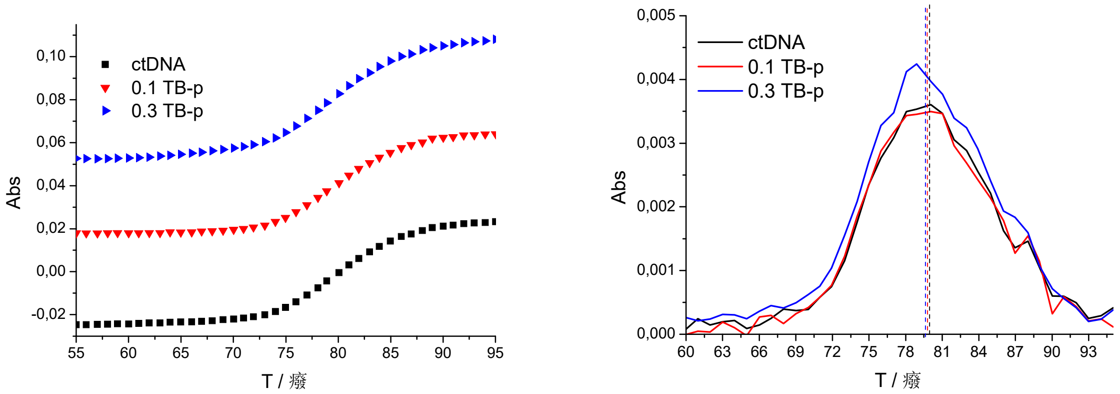

a) **b**)

Figure S9. a) Melting curve of ctDNA upon addition *r* = 0.1 and *r* = 0.3 ([compound / [polynucleotide]) of **TB-p** at pH 7.0 (buffer sodium cacodylate, *I* = 0.05 M); **b)** first derivation of absorbance on temperature.

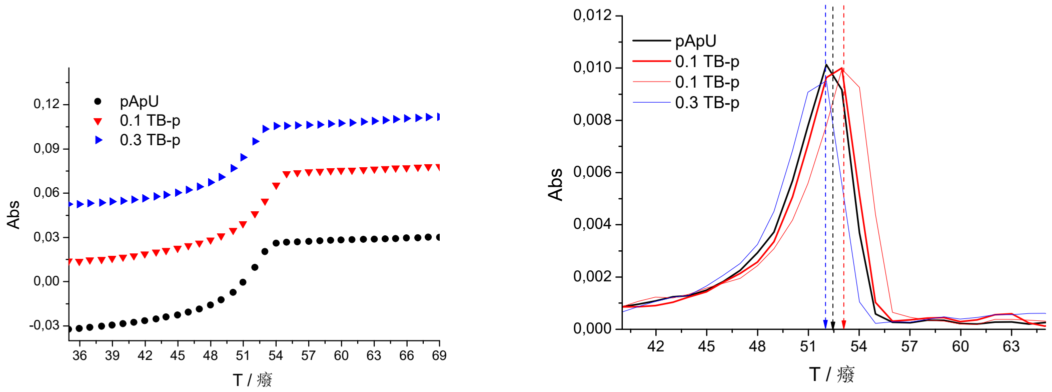

a) b)

Figure S10. a) Melting curve of pApU upon addition *r* = 0.1 and *r* = 0.3 ([compound / [polynucleotide]) of **TB-p** at pH 7.0 (buffer sodium cacodylate, *I* = 0.05 M); **b)** first derivation of absorbance on temperature.

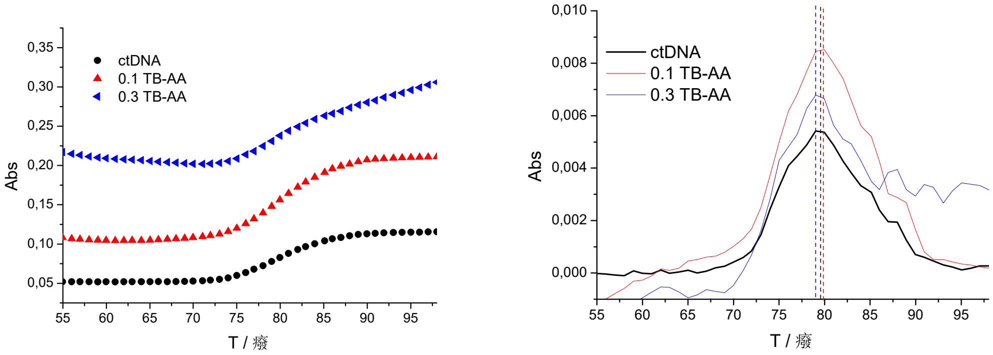

a) b)

Figure S11. a) Melting curve of ctDNA upon addition r = 0.1 and r = 0.3 ([compound / [polynucleotide]) of **TB-AA** at pH 7.0 (buffer sodium cacodylate, I = 0.05 M); **b)** first derivation of absorbance on temperature.

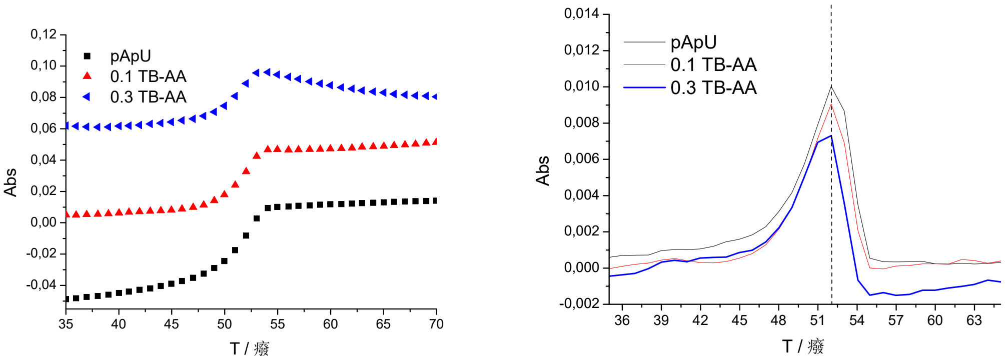

a) b)

Figure S12. a) Melting curve of pApU upon addition r = 0.1 and r = 0.3 ([compound / [polynucleotide]) of **TB-AA** at pH 7.0 (buffer sodium cacodylate, I = 0.05 M); **b)** first derivation of absorbance on temperature.

Table S2. ΔT_m - Values[a] (°C) for different ratios [b]***r*** of **TB-p and TB-AA** added to polynucleotide.

	[b]***r***	**ctDNA**	**pApU**
**TB-p**	0.1	-0.6	1.0
	0.3	-1.2	0
**TB-AA**	0.1	0	0
	0.3	-0.5	0

[a] ΔT_m-Values
[b] r = [compound] / [polynucleotide]

4. Circular dichroism (CircD) experiments

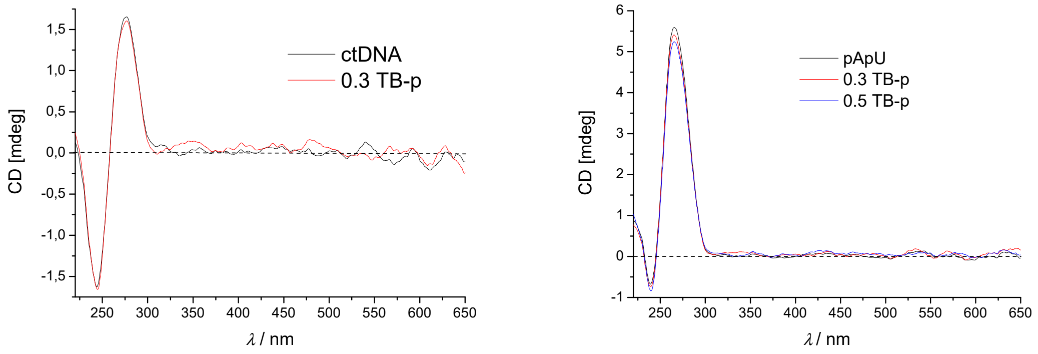

a) **b**)

Figure S13. CD titration of **a**) ctDNA, **b**) pApU ($c = 2 \times 10^{-5}$ M) with **TB-p** at molar ratio r = [compound] / [polynucleotide] (pH 7.0, buffer sodium cacodylate, I = 0.05 M).

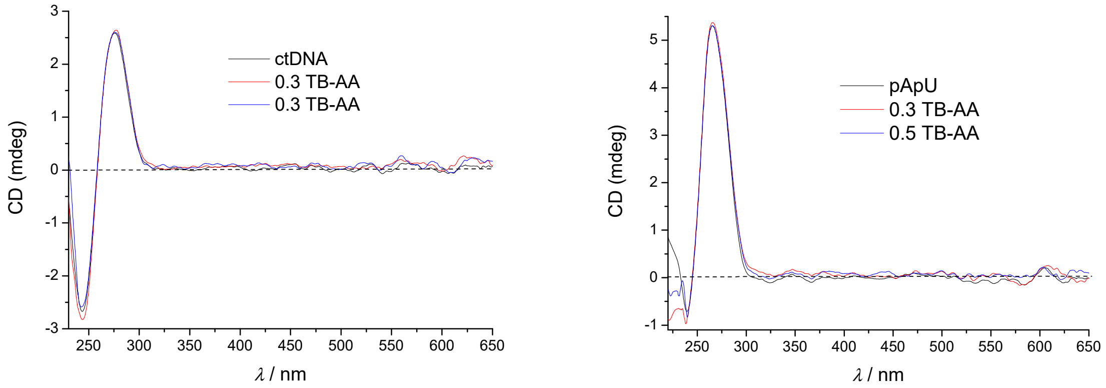

a) **b**)

Figure S14. CD titration of **a**) ctDNA, **b**) pApU ($c = 2 \times 10^{-5}$ M) with **TB-AA** at molar ratio r = [compound] / [polynucleotide] (pH 7.0, buffer sodium cacodylate, I = 0.05 M).

5. Interactions with DNA, RNA, BSA

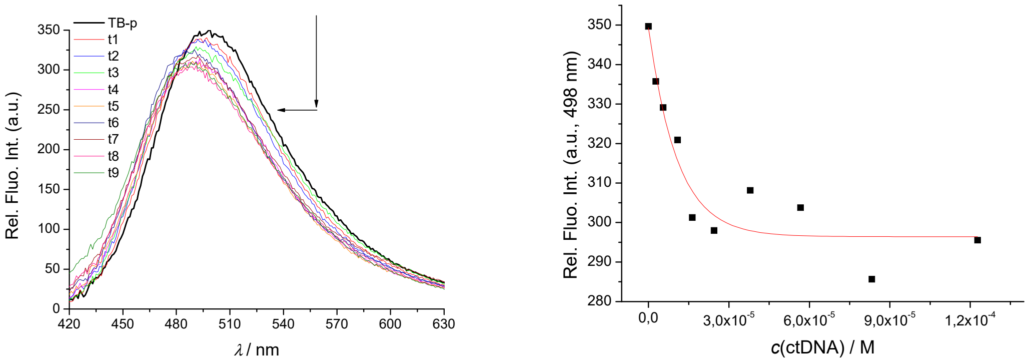

a) b)

Figure S15. a) Fluorimetric titration of **TB-p** (c = 5.00 × 10^{-6} M) on λ_{exc} = 387 nm with ctDNA; **b)** Dependence of **TB-p** intensity at λ_{max} = 498 nm on c(ctDNA), at pH 7.0, sodium cacodylate buffer, I = 0.05 M.

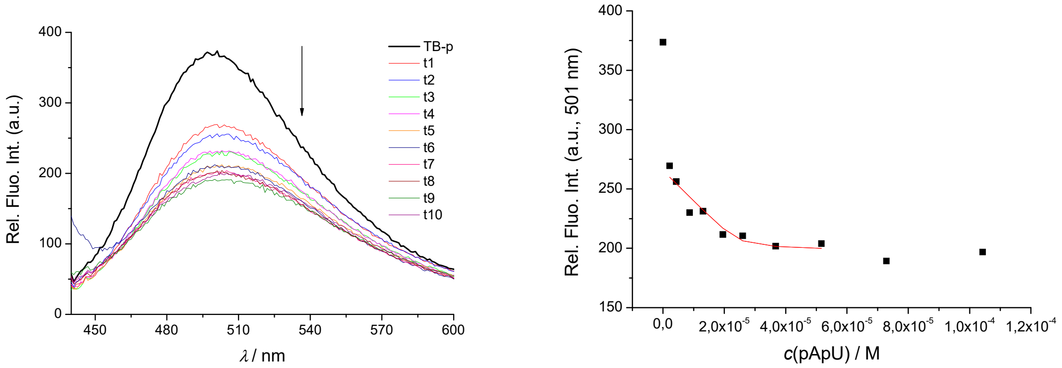

a) b)

Figure S16. a) Fluorimetric titration of **TB-p** (c = 5.0 × 10^{-6} M) on λ_{exc} = 387 nm with pApU; **b)** Dependence of **TB-p** intensity at λ_{max} = 501 nm on c(pApU), at pH 7.0, sodium cacodylate buffer, I = 0.05 M.

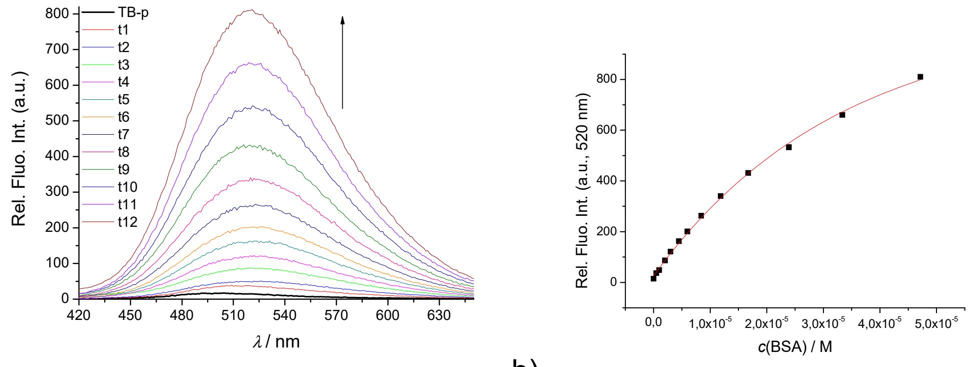

a) b)

Figure S17. a) Fluorimetric titration of **TB-p** (c = 5.00 × 10^{-6} M) on λ_{exc} = 387 nm with BSA; **b)** Dependence of **TB-p** intensity at λ_{max} = 520 nm on c(BSA), at pH 7.0, sodium cacodylate buffer, I = 0.05 M.

6. Interactions of TB-AA with cyclodextrins

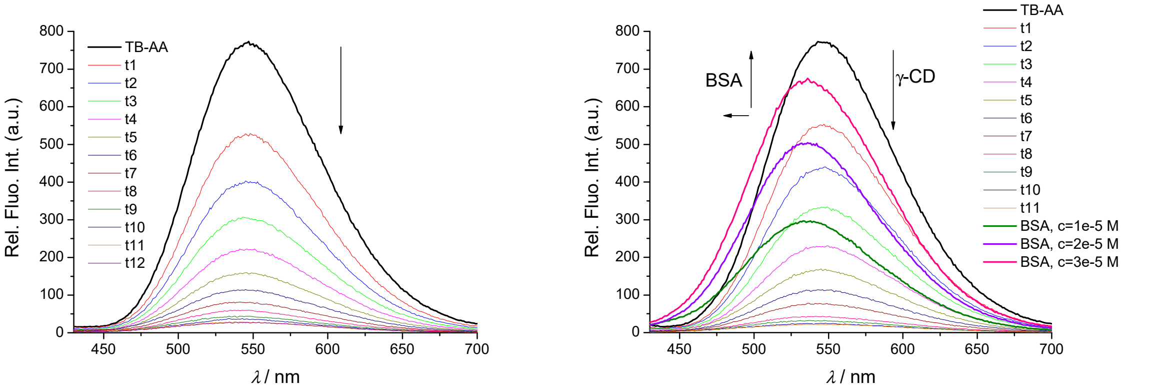

a) **b**)

Figure S18. Fluorimetric titration of **TB-AA** (c = 3.00 × 10^{-6} M) on λ_{exc} = 407 nm with **a**) β-cyclodextrin; **b**) γ-cyclodextrin and competition with BSA, at pH 7.0, sodium cacodylate buffer, I = 0.05 M.

7. NMR and HRMS data

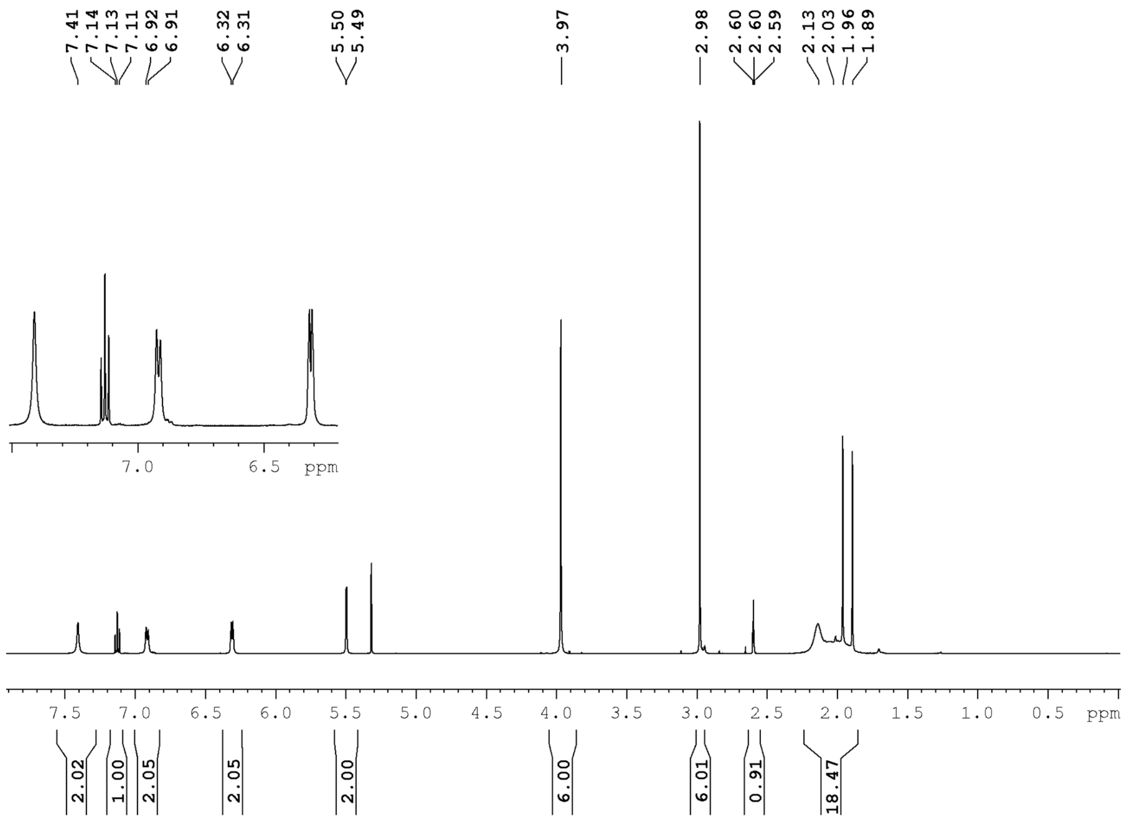

Figure S19. ^{1}H NMR spectrum of **TB-p** in CD_2Cl_2 at 500 MHz.

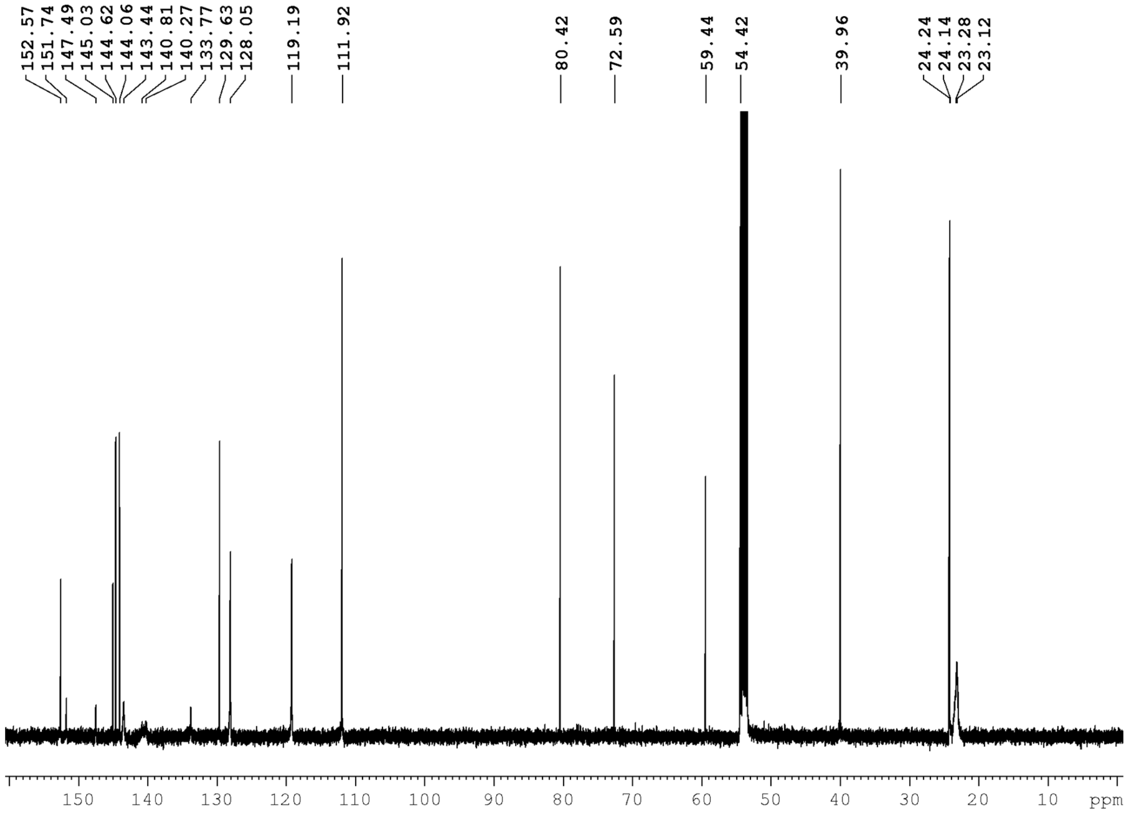

Figure S20. $^{13}C\{^1H\}$ NMR spectrum of **TB-p** in CD_2Cl_2 at 125 MHz.

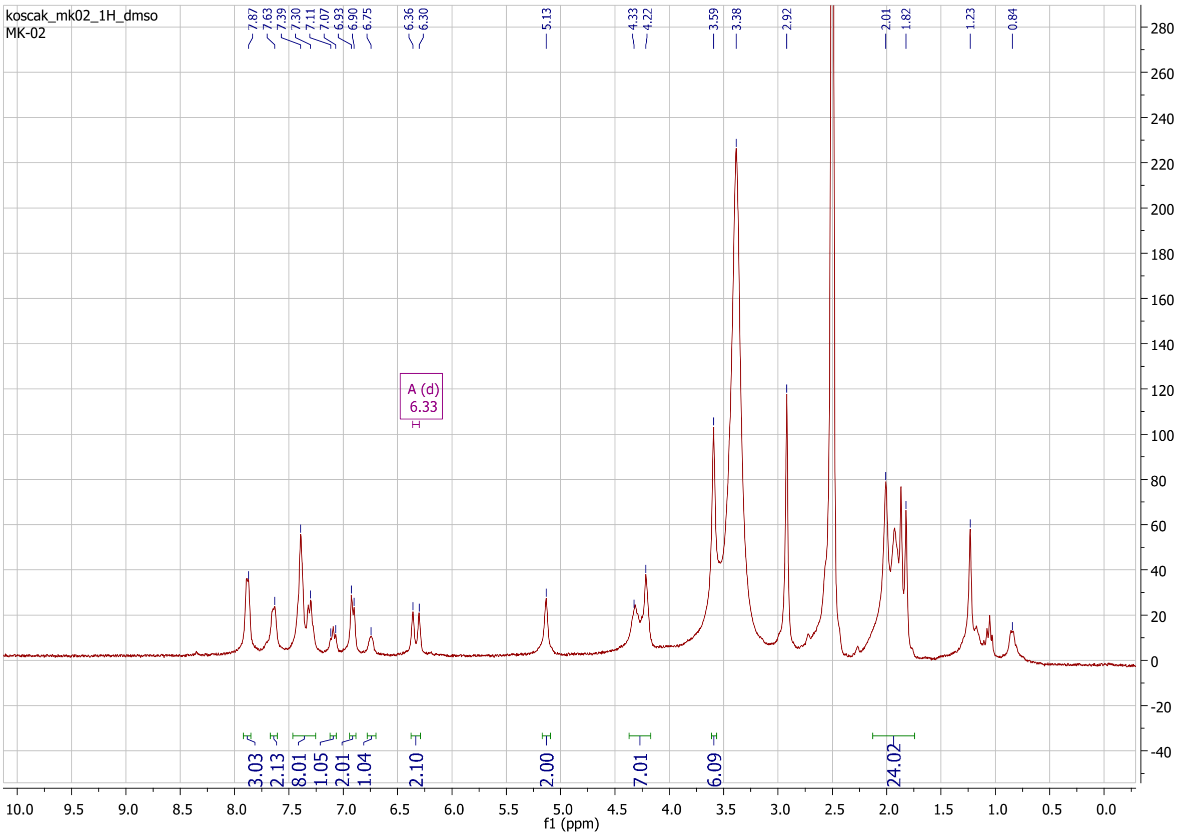

Figure S21. ^{1}H NMR spectrum of **TB-AA** in DMSO at 300 MHz.

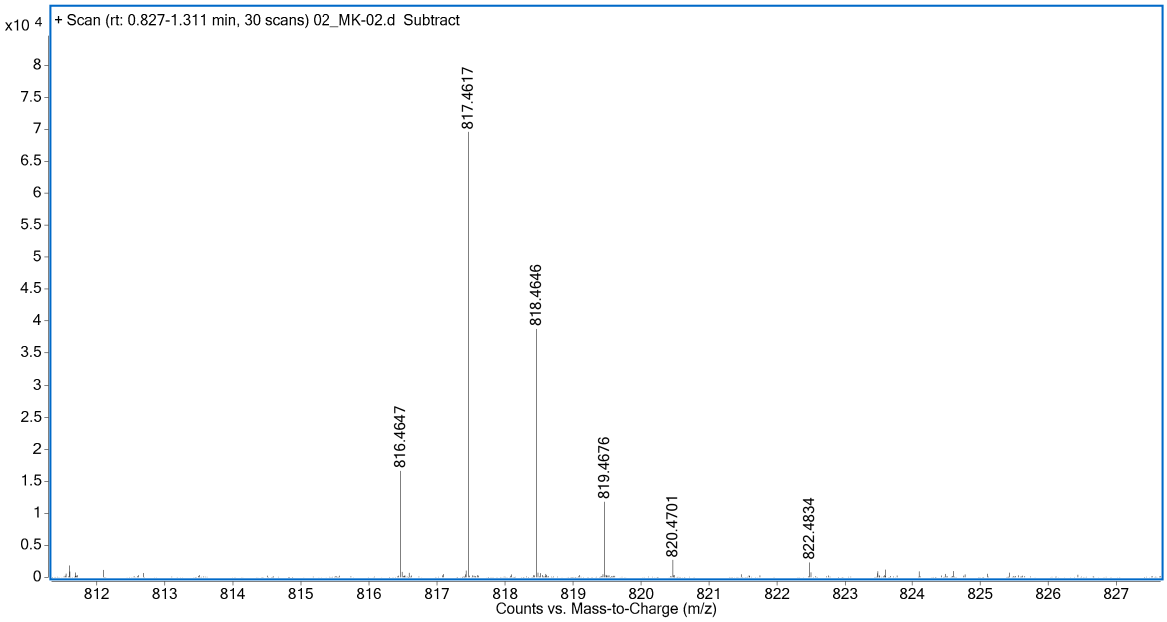

Figure S22. HRMS analysis of **TB-AA** in MeOH at LC/Q-TOF.
